# Contextual Risk Factors for Low Birth Weight: A Multilevel Analysis

**DOI:** 10.1371/journal.pone.0109333

**Published:** 2014-10-31

**Authors:** Gbenga A. Kayode, Mary Amoakoh-Coleman, Irene Akua Agyepong, Evelyn Ansah, Diederick E. Grobbee, Kerstin Klipstein-Grobusch

**Affiliations:** 1 Julius Global Health, Julius Center for Health Sciences and Primary Care, University Medical Centre Utrecht, Utrecht, The Netherlands; 2 Ghana Health Service, Greater Accra Region, Accra, Ghana; 3 School of Public Health, University of Ghana, Legon, Accra, Ghana; 4 Division of Epidemiology and Biostatistics, School of Public Health, Faculty of Health Science, University of Witwatersrand, Johannesburg, South Africa; University of Ottawa, Canada

## Abstract

**Background:**

Low birth weight (LBW) remains to be a leading cause of neonatal death and a major contributor to infant and under-five mortality. Its prevalence has not declined in the last decade in sub-Saharan Africa (SSA) and Asia. Some individual level factors have been identified as risk factors for LBW but knowledge is limited on contextual risk factors for LBW especially in SSA.

**Methods:**

Contextual risk factors for LBW in Ghana were identified by performing multivariable multilevel logistic regression analysis of 6,900 mothers dwelling in 412 communities that participated in the 2003 and 2008 Demographic and Health Surveys in Ghana.

**Results:**

Contextual-level factors were significantly associated with LBW: Being a rural dweller increased the likelihood of having a LBW infant by 43% (OR 1.43; 95% CI 1.01–2.01; P-value <0.05) while living in poverty-concentrated communities increased the risk of having a LBW infant twofold (OR 2.16; 95% CI 1.29–3.61; P-value <0.01). In neighbourhoods with a high coverage of safe water supply the odds of having a LBW infant reduced by 28% (OR 0.74; 95% CI 0.57–0.96; P-value <0.05).

**Conclusion:**

This study showed contextual risk factors to have independent effects on the prevalence of LBW infants. Being a rural dweller, living in a community with a high concentration of poverty and a low coverage of safe water supply were found to increase the prevalence of LBW infants. Implementing appropriate community-based intervention programmes will likely reduce the occurrence of LBW infants.

## Background

Deliveries in low and middle income countries are often complicated by adverse birth outcomes such as stillbirth, early neonatal mortality and morbidity. Low birth weight (LBW) remains to be a leading cause of neonatal death [1], and is a major contributor to infant and under-five mortality [2]. Infants weighing less than 2500 grams at birth are regarded as LBW infants. LBW is associated with early and late morbid conditions such as coronary heart disease [3], [4], non-insulin dependent diabetes [5], childhood hypertension [6], behavioural disorders [7], impaired cognitive function [8], [9], psychological disorders [10], and these usually have long-term financial burden [11].

Infants can have LBW either as a result of small-for-gestational-age (SGA) or preterm delivery. An infant is said to be small-for-gestational-age when the gender-specific birth weight is below the 10^th^ percentile for the appropriate gestational age [12], [13]; such a condition could be constitutional or pathological, in the latter case, it is referred to as Intrauterine Growth Retardation (IUGR). Due to the devastating public health implications of LBW, the United Nations incorporated LBW into its action plans and aimed to reduce its incidence by one-third by 2015 [14].

Globally, one out of seven infants is born with LBW. The incidence has not declined in the last decade in Sub-Saharan Africa (SSA) and Asia [15]; even in Europe only very few countries have reduced its incidence [16]. In Ghana, the recent incidence of LBW infants was estimated at 160 per 1000 births and has not witnessed any reduction in the last decade [17]. The aetiology of LBW is yet to be completely understood even though several studies have attempted to unravel the underlying causes. Constitutional factors such as sex [18], maternal height [19] and weight [20] have been identified as risk factors for LBW. Similarly, maternal health, demographic and nutritional factors which include maternal age [21], ethnicity [22], parity [23], [24], birth interval [18], multiple gestation [21], maternal comorbidity [24], skilled antenatal care [21], [25], placenta causes [26], nutritional deficiencies [18], and body mass index [19] have been linked with LBW. In addition, maternal socioeconomic and psychological factors which comprise education [18], alcohol intake [27], smoking [24], use of hard drugs [28], occupation [29], wealth status [18], marital status [22], and domestic violence [30] were also found to be associated with LBW.

However, despite these numerous identified individual-level risk factors for LBW, there is a wide gap in epidemiological knowledge regarding the common effect that the community context has on the incidence of LBW especially in SSA. Studies conducted outside SSA have identified the impact of residential segregation [31], [32], neighbourhood poverty [31], [32] and unemployment on LBW [29] but up to date, the contextual effect on LBW has not been adequately investigated in SSA even though emerging evidence from Nigeria showed that contextual factors have an impact on under-five mortality and morbidity [33]–[35]. Contextual effects can best be revealed by disentangling the effect of contextual factors from individual factors using a multilevel regression model. It allows the incorporation of contextual factors into the regression model to prevent a residual confounding effect of omitting contextual factors, overestimation of the association, and underestimation of the standard error [36]. Identifying contextual factors for LBW will guide the development of community-based interventions aiming to reduce the occurrence of LBW. Thus, this study aimed to identify contextual risk factors for LBW in Ghana.

## Methods

### Study design

This is a population-based study that utilized a combined dataset of the 2003 and 2008 Ghana Demographic and Health Survey (GHDS) to identify contextual risk factors for LBW in Ghana.

### Data collection

Comprehensive information on the sampling techniques and procedures for the GDHS data collection have been published elsewhere [37], [38]. Detailed information on all under-five children in the last five years was captured in both surveys and 12,474 households, 11,045 women and 10,114 men were identified for interviews. Face-to-face interviews were conducted for all women aged 15 to 49 years and men aged 15 to 59 years in the sampled households by use of questionnaires covering socioeconomic, demographic and health indicators.

### Variables

#### Outcome

Mothers were asked to recall the birth weight of their infants or provide hospital cards to confirm it. In case they could neither recall the birth weight of their infants nor provide a hospital card, they were asked whether the birth weight of their babies was very big, bigger than average, average, smaller than average or very small. For the purpose of the current analysis we classified infants with a birth weight smaller than average and very small as LBW infants [37], [38].

#### Contextual-level factors (determinants)

We referred to the primary sampling unit (PSU) of the DHS data as a community. The impact of the community context on low birth weight was examined by considering place of residence (rural/urban), proportion of the community that were having access to healthcare and safe water coverage, and proportion of illiterate (those that can neither read nor write in any language) and those living in extreme poverty in the community (estimated asset index <20% poorest quintile) as contextual factors.

#### Individual-level factors (potential confounders)

Un-confounded effects of contextual risk factors on LBW were estimated after considering potential confounders based on epidemiological knowledge, prior studies, and the available information in the GDHS. Maternal age, parity, birth interval, unplanned pregnancy, ethnicity, anaemia in pregnancy, use of antenatal care, use of antimalarial or mosquito nets during pregnancy, smoking, body mass index, maternal education, occupation, wealth status and marital status were considered as potential confounders in the analysis. Marital status was classified as currently, formerly and never married. Maternal educational attainment was categorized into no education, primary, and secondary or higher education. The GDHS applied an asset-based approach to estimate household wealth status [39], similar to previous studies conducted [40], [41].

### Statistical analysis

#### Descriptive analyses

In the descriptive analyses, the characteristics of the study population were expressed in terms of numbers and percentages. The prevalences of LBW across the categories of the explanatory variables were estimated in terms of numbers and percentages.

#### Statistical modeling

We applied a two-level multivariable multilevel logistic regression analysis, fitting three models different models. Model 1 (empty or null model) has no explanatory variable and we used it to decompose the total variance of LBW between the contextual and individual level. Model 2 contained the contextual-level factors and we extended this model to form model 3 by accommodating all the potential confounders (individual-level factors). Sensitivity analysis was conducted to assess whether the results of the analyses were consistent with the group of LBW infants classified to be of very small birth weight. This was necessitated by the potential risk of having a misclassified outcome by maternal self-report.

#### Measures of association (fixed effects)

Measures of association between the contextual risk factors and LBW were reported in terms of odds ratios (OR) with their P-values and 95% confidence interval (CI) after considering potential confounders.

#### Measures of variation (random effects)

Random effects were expressed in terms of Area variance (AV), Median Odds Ratio (MOR) and Intra-Cluster Correlation (ICC)/Variance Partition Coefficient (VPC).

#### Model fitness & precision

The fitness of the model was assessed using Akaike Information Criterion (AIC) while Variance Inflation Factor(VIF) was used to check for multicollinarity in the model. Two-tailed Wald test at significance level of alpha equal to 5% was used to determine the statistical significance of the determinants and all the analyses were performed with StataSE 11 software package, StataCorp LP, Texas, United States.

### Ethical approval

Ethical clearance to conduct GDHS was obtained from the Ethics Review Committee, Ghana Health Service, Accra, Ghana and the Ethics Committee of ICF Macro in Calverton, United States. GDHS data are public access data and were made available to us upon request by Measure DHS.

## Results

### Population characteristics

Table 1 shows the descriptive characteristics of the 6,900 women aged 15–49 years, dwelling in 412 different communities who participated in the Ghana demographic and health survey (GDHS) on child health in the last decade. Characteristics of women between the 2003 and 2008 GHDS did not differ significantly, thus both surveys were combined and analysed. About two-fifths of the women interviewed were illiterate and had an unwanted pregnancy. More than half of them were living in poverty and a quarter of them were either obese or over weight. The majority of the women were non-smokers, cohabiting with their husband and almost one-sixth of them had LBW infants. The prevalence of LBW was estimated at 16.9 percent, and observed to be higher among rural dwellers, and in communities with low coverage of safe water supply, poor access to healthcare, a high proportion of illiterates and those living in extreme poverty.

**Table 1:** General characteristics of the study population, GDHS 2003 and 2008.

INDIVIDUAL-LEVEL DETERMINANTS (POTENTIAL COFOUNDERS)				
		Low Birth Weight		
	Number (%)	Yes	No	
	n (%)	n (%)	n (%)	Total N (%)
**Maternal Age**				
*15–24 years*	1,537 (23)	300 (20)	1,221 (80)	1,521 (100)
*25–34 years*	3,300 (48)	533 (16)	2,722 (84)	3,255 (100)
*35–49 years*	1,999 (29)	309 (16)	1,664 (84)	1,973 (100)
**Marital status**				
Never married	227 (3)	42 (19)	185 (81)	227 (100)
Currently married	6,224 (91)	1,027 (17)	5,117 (83)	6,144 (100)
Formerly married	385 (6)	73 (19)	305 (81)	378 (100)
**Maternal education**				
*No education*	2,956 (43)	555 (19)	2,353 (81)	2,908 (100)
*Primary*	1,545 (23)	263 (17)	1,266 (82)	1,529 (100)
*Secondary or higher*	2,335 (34)	324 (14)	1,988 (86)	2,312 (100)
**Maternal occupation**				
*Unemployed*	689 (10)	118 (17)	558 (83)	676 (100)
*Manual*	4,184 (62)	760 (18)	3,377 (82)	4,137 (100)
*Skilled worker*	1,922 (28)	256 (14)	1,640 (86)	1,896 (100)
**Parity**				
One	1,035 (15)	206 (20)	821 (80)	1,027 (100)
Two – four	3,514 (51)	538 (16)	2,933 (84)	3,471 (100)
Five and above	2,287 (34)	398 (18)	5,607 (83)	6,749 (100)
**Birth interval**				
* <18 months*	227 (4)	40 (18)	181 (82)	221 (100)
* 18–36 months*	2,060 (39)	329 (16)	1,706 (84)	2,035 (100)
* >36 months*	3,016 (57)	469 (16)	2,508 (84)	2,977 (100)
**Skilled antenatal care**				
Yes	4,458 (94)	703 (16)	3,741 (84)	4,444 (100)
No	293 (6)	76 (25)	217 (74)	293 (100)
**Body mass index (Kg/m^2^)**				
Underweight	536 (8)	109 (21)	410 (79)	528 (100)
Normal weight	4,545 (67)	794 (18)	3,695 (82)	4,489 (100)
Over weight	1,116 (16)	150 (14)	951 (86)	1,101 (100)
Obese	639 (9)	89 (14)	542 (86)	631 (100)
**Maternal smoking**				
No	6,825 (99.9)	1,139 (17)	5,600 (83)	6,739 (100)
Yes	8 (0.1)	2 (29)	5 (71)	7 (100)
**Use of mosquito net or malaria prophylaxis**				
*No*	2,805 (41)	533 (19)	2,209 (81)	2,742 (100)
*Yes*	4,031 (59)	609 (15)	3,398 (85)	4,007 (100)
**Maternal anaemia**				
*Severe*	81 (1)	12 (15)	68 (85)	80 (100)
*Moderate*	881 (13)	151 (17)	721 (83)	872 (100)
*Mild*	2,594 (40)	472 (18)	2,090 (82)	2,562 (100)
*Not anaemic*	3,012 (46)	457 (15)	2,514 (85)	2,971 (100)
**Index pregnancy wanted**				
*Wanted then*	3,978 (58)	637 (16)	3,296 (84)	3,933 (100)
*Not wanted*	1,752 (26)	195 (18)	885 (82)	1,080 (100)
*Wanted later*	1,094 (16)	310 (18)	1,426 (82)	1,736 (100)
**Wealth index**				
*Poor*	3,773 (55)	683 (18)	3,046 (82)	3,729 (100)
*Average*	1,186 (18)	206 (18)	962 (82)	1,168 (100)
*Rich*	1,877 (28)	253 (14)	1,599 (86)	1,852 (100)
**Ethnicity**				
Akan	2,612 (38.2)	403 (16)	2,181 (84)	2,584 (100)
*Ga/Guan*	578 (8)	81 (14)	490 (86)	571 (100)
*Ewe*	791 (12)	113 (14)	671 (86)	784 (100)
*Mole-dagbani*	1,697 (25)	292 (18)	1,374 (82)	1,666 (100)
*Grussi/Gruma*	703 (10)	147 (21)	550 (79)	697 (100)
*Others*	451 (7)	106 (24)	337 (76)	443 (100)
**VARIABLES USED TO OPERATIONALISED POPULATION-LEVEL FACTORS**				
**Residence**				
Rural	4,793 (70)	868 (18)	3,863 (82)	4,731 (100)
Urban	2,043 (30)	274 (14)	1,744 (86)	2,018 (100)
**Access to healthcare**				
Difficult	2,650 (39)	484 (19)	2,129 (81)	2,613 (100)
Not difficult	4,175 (61)	658 (16)	3,469 (84)	4,127 (100)
**Water source**				
Safe	3,115 (51)	453 (15)	2,618 (85)	3,071 (100)
Not safe	2,992 (49)	554 (19)	2,403 (81)	2,957 (100)
**Extreme poverty**				
Yes	1,353 (20)	295 (22)	1,036 (78)	1,331 (100)
No	5,483 (80)	847 (16)	4,571 (84)	5,418 (100)
**Illiterate**				
Yes	2,956 (43)	555 (19)	2,353 (81)	2,908 (100)
No	3,880 (57)	587 (15)	3,254 (85)	3,841 (100)

### Random effects (measures of variation)

The results of the multivariable multilevel logistic regression (MMLR) are shown in Table 2. In model 1 (null or empty model), variance component analysis was performed to decompose the total variance of LBW and estimate the contextual-level variance which indicates the total variance of LBW that can be attributed to the context of the community in which the mothers were dwelling. The applicability of MMLR in the analysis was justified by the significance of the contextual-level variance [area variance (AV)  = 0.208; standard error (SE)  = 0.048; P-value  = <0.001], indicating the existence of significant differences between communities with regard to LBW incidence. The AV was expressed as intracluster correlation (ICC) and median odds ratio (MOR); the ICC was 0.060 which implied that 6% of the total variance of LBW in Ghana can be attributed to the context of the community where the mothers were living. The MOR was 1.54 (95% C.I 1.41–1.72) which showed that the likelihood of having a LBW increased by 54% when mothers moved from low to high risk neighbourhoods.

**Table 2:** Associations between low birth weight and contextual risk factors, GDHS 2003 and 2008.

	Null model	Mode with population level factors	Mode with individual & community level determinants
**FIXED EFFECT (OR, 95% CI, P-value)**			
**Contextual-level factors**			
**Residence**			
Rural		1.17 (0.96–1.42)	1.43 (1.01–2.01)*
Urban		1 (reference)	1 (reference)
**Community poverty level**			
High		1.61 (1.13–2.29)**	2.16 (1.29–3.61)**
Low		1 (reference)	1 (reference)
**Community Illiteracy level**			
High		1.13 (0.83–1.54)	1.22 (0.70–2.12)
Low		1 (reference)	1 (reference)
**Community safe water coverage**			
High		0.78 (0.65–0.93)**	0.74 (0.57–0.96)*
Low		1 (reference)	1 (reference)
**Community healthcare access**			
Difficult		1.09 (0.84–1.42)	1.28 (0.87–1.87)
Not difficult		1 (reference)	1 (reference)
**RANDOM EFFECT**			
**Area Variance (SE)**	0.208 (0.048)***	0.190 (0.047)***	0.168 (0.081)**
**PCV**		−8.7%	−12.1%
**MOR**	1.54 (1.41–1.72)	1.51 (1.38–1.70)	1.48 (1.28–1.87)
**ICC (latent variable method)**	0.060	0.055	0.049
**AIC**	6098.007	6059.623	2885.930

Model 1 is the null model, contained no explanatory variable.

Model 2 adjusted for contextual-level characteristics.

Model 3 adjusted for both population-level and individual-level characteristics.

Individual-level characteristics adjusted for: maternal age, marital status, parity, maternal BMI, maternal education, maternal occupation, birth interval, use of mosquito net or malaria prophylactic, anaemia in pregnancy, antenatal care, smoking, unwanted pregnancy, maternal nutritional intake, ethnicity & wealth index.

Abbreviations: OR: odds ratio; SE: standard error; PCV: proportional change in variance; 95% CI: 95% confidence interval; MOR: median odds ratio; ICC: intracluster correlation.

***p<0.001, **p<0.01, and *p<0.05.

After extending Model 1 to form Model 2 by entering the contextual risk factors, AV (AV 0.190; SE 0.047; P-value <0.001), MOR 1.51 (95% CI 1.38–1.70) and ICC (0.055) remained significant but reduced because part of the contextual-level variance was explained by the contextual risk factors in the model. The estimated proportional change in variance (PCV) was −8.7%, indicating that 8.7% of the contextual-level variance was explained by the contextual risk factors entered into the model. Further, in order to estimate an un-confounded effect of the contextual risk factors, we adjusted for the potential confounders (individual-level factors) in Model 3. The AV (AV 0.167; SE 0.081; P-value <0.01), MOR 1.47 (95% CI 1.27–1.70) and ICC (0.048) remained significant but reduced and the PCV was −12.1%, meaning that 12.1% of the contextual-level variance of LBW can be explained by the compositional characteristics of mothers dwelling in the communities. About 4.8% of the total variance of LBW that can be attributed to the contextual-level factors remained significant even after considering some contextual risk factors for LBW.

### Fixed effect (measures of association)

The contextual risk factors for LBW that remained significant after adjusting for the potential confounders (individual-level factors) are shown in Table 2. Being a rural dweller increased the likelihood of having a LBW infant by 43% (OR 1.43; P-value <0.05; 95% CI 1.01–2.01). Similarly, dwelling in a community with a high proportion of people living in extreme poverty increased the likelihood of having a LBW infant by twofold (OR 2.16; P-value <0.01; 95% CI 1.29–3.61) while residing in a community with a high level of safe water coverage reduced the odds of having an infant with LBW by 28% (OR 0.74; P-value <0.05; 95% CI 0.57–0.96).

### Model fit statistics

There were progressive reductions in AIC from Model 1 to 3, indicating that the explanatory value of the model increases from Model 1 to 3. In other words Model 3 explained the determinants better than Model 1 and 2.

### Sensitivity analysis

To assess the potential effect of misclassification of low birth weight on the association observed between contextual-level factors and low birth weight we limited our analyses to the infants considered to be of very small birth weight (VLBW). Area variance was observed to increase and remained significant (0.534; SE 0.127), the intra-cluster correlation (ICC 0.140) and median odds ratio (MOR 2.00, 95% CI 1.73–2.40) also increased. The odds of having a VLBW infant increased four-fold (OR 4.02; P-value <0.01; 95% CI 1.72–9.38) among mothers living in communities with a high concentration of extreme poverty while mothers living in communities with a high coverage of safe water supply reduced their likelihood of having a LBW infants by 46% (OR 0.54; P-value <0.05; 95% CI 0.40–0.90) compared to their counterparts dwelling in areas with a low coverage of safe water supply. However the statistical significant effect of place of residence on LBW was deattenuated (OR 1.33; P-value >0.1; 95% CI 0.77–2.29).

## Discussion

### Main findings

This study investigated LBW beyond the traditional method of examining the risk factors for LBW by estimating the association between the context of the community where the mothers were residing, and the prevalence of LBW after controlling for individual characteristics of the mothers. The study showed contextual factors to be significantly associated with LBW. Being a rural dweller increased the likelihood of having a LBW infant and the plausible explanation for this is that living in rural areas in SSA simply means residing in a deprived community in terms of job opportunities, social amenities and infrastructures which carries an increased risk of LBW. This finding is consistent with a previous study conducted in the United States that found that mothers residing in urban areas tend to be protected from having a LBW infant [42]. However, studies from Brazil reported urbanization to be associated with increased risk of having a LBW infant [43], [44]. Authors have likened this relationship to “low birth weight epidemiological paradox” found in Mexican-America mothers, which to date has not been observed in a SSA context.

Further, residing in wealthier communities was observed to protect women from having LBW infants compared to their counterparts dwelling in neighbourhoods with a high concentration of extreme poverty. Dwelling in such contexts might lead to maternal psychosocial stress which in turn has been implicated to increase the release of catecholamine and cortisol, and subsequent stimulate the release of corticotrophin releasing hormone (CRH) through cortisol. The release of CRH has been hypothesized to initiate the onset of labour [45] via a series biochemical processes while cortisol has been linked with IUGR [46]. This relationship is in line with observations of previous studies [32], [47]. Mothers living in a neighbourhood with a low coverage of safe water were observed to have LBW infants more often than those dwelling in a neighbourhood with a high coverage of safe water supply. The most likely explanation for this is that unsafe water supply will increase episodes of gastrointestinal infections during pregnancy which could impair normal fetal development or initiate preterm labour. This finding is in accordance with a prior study conducted in the United Kingdom that showed that elevated concentrations of disinfection by-product in drinking water increased the risk of LBW [48].

Considering the outcomes of this study, implementation of community-based intervention programs that can bridge the gaps between the rural and urban settlements in terms of infrastructural development are considered to be necessary. In the absence of any intervention, poverty can become an inter-generational problem that may be difficult to address. Thus, both government and non-governmental organizations will need to be more proactive towards implementing sustainable population-based poverty eradication programs coupled with women and youth empowerment programs. Provision of regular safe water supply to the communities will likely reduce the occurrence of LBW. Impact of such programs will go beyond individual-level, population will be its unit of manifestation. It is important to note that 4.8% of the variance in LBW in Ghana that was attributed to contextual factors remained significant even after considering contextual-level factors indicating why it is important for future studies to identify other contextual risk factors for LBW.

### Study limitations and strengths

As we used nationally representative data with excellent individual and household response rates for this study, study findings can easily be generalized for Ghana and beyond. Likewise the application of multilevel analysis in this study, made it possible to disentangle the effects of the individual and contextual factors on LBW. To the best of our knowledge, none of the previous multilevel studies on LBW accounted for haemoglobin concentration status and the desire to be pregnant unlike our study where these factors and other known potential confounders were considered.

However, limitations of this study cannot be overlooked. Alcohol intake was not captured in the GDHS so it was not included as a potential confounder but we believe this will not have any significant impact on the observed contextual effects because smoking and alcohol intake among women in Ghana is rare; for instance this study found that the prevalence of smoking among mothers was 0.1%. Because we examined secondary cross-sectional data, we were unable to evaluate the effect of the duration of living in a poverty-concentrated community on LBW. This study did not examine for possible cross-level interaction effect, thus we suggest that subsequent study should explore this area.

Mothers that neither had any evidence to confirm the birth weight of their children nor were able to recollect the birth weight were asked to specify whether the birth weight of their children was very big, bigger than average, average, smaller than average or very small. We considered smaller than average and very small as LBW in our analysis. Every mother was given the opportunity to specify the birth weight of their baby thus missing data is not an issue in this variable. The main challenge here is the possibility of misclassifying birth weight by mothers that could not provide the birth weight of their children due to their inability to remember the birth weight or the birth weight was not measured at birth; this is different from recall bias. Thus misclassification of birth weight could have occurred, however previous results from demographic and health survey data from Cambodia, Kazakhstan and Malawi assessing whether mother's perception of a baby's birth weight is a good proxy for birth weight noted good agreement between mother's perception of a baby's birth weight and measured birth weight [49], [50]. Comparison of the prevalence of low birth weight in our study (16.9%) to the prevalence of low birth weight in another Ghanaian data source support this notion [17].

Further based on logical reasoning out of the five classes of birth weight (very big, bigger than average, average, smaller than average or very small) the possibility of misclassifying infants with normal birth weight as low birth weight should be minimal among infants classified as “very small infants”. Indeed, the observed difference in the prevalence of “very small infant” among infants that their birth weight was provided and those that were assessed based on mother's perception of a baby's size at birth was less than 1.8%. We thus, examined whether the contextual effects observed remained significant in the subgroup of infants classified to be very small, i.e. very low birth weight (VLBW). The random effect of the community context increased (AV 0.534, SE 0.127; MOR 2.00, 95% CI 1.73–2.40; ICC 0.140) Likewise the effect of poverty (OR 4.02; P-value <0.01; 95% CI 1.72–9.38) and safe water coverage (OR 0.54; P-value <0.05; 95% CI 0.40–0.90) were more pronounced when VLBW infants were considered; reaffirming the importance of these contextual level factors on the occurrence of LBW.

## Conclusions

This study has demonstrated that contextual risk factors have independent effects on the prevalence of LBW infants in Ghanaian communities regardless of individual-level characteristics of the mothers. Being a rural dweller, Living in a community with a high concentration of poverty and a low coverage of safe water supply were found to be associated with a high prevalence LBW while poverty and poor coverage of safe water showed a pronounced impact on the prevalence of VLBW. Implementing community-based intervention programs that will address poverty alleviation, provision of regular safe water supply and the infrastructural development of rural communities will likely reduce the occurrence of LWB.
